# In Vitro Inhibitory Activity of Three Hospital Biocides Against Multidrug-Resistant Clinical Isolates: A Pilot Study From Morocco

**DOI:** 10.7759/cureus.114655

**Published:** 2026-08-17

**Authors:** Elmostafa Benaissa, Zakaria Amal, Najoua Hnach, Yassine Ben Lahlou, Mariama Chadli

**Affiliations:** 1 Bacteriology, Mohammed V Military Training Hospital, Rabat, MAR; 2 Clinical Bacteriology, Mohammed V Military Training Hospital, Rabat, MAR; 3 Epidemiology and Public Health, Faculty of Medicine and Pharmacy of Rabat, Rabat, MAR

**Keywords:** biocides, disinfectants, ethanol, hydrogen peroxide, multidrug-resistant bacteria, sodium hypochlorite

## Abstract

Background

Biocides are central to infection prevention and control, but incorrect preparation or dilution may reduce antimicrobial activity. This exploratory pilot study evaluated visible-growth inhibition produced by three hospital biocide working solutions against selected multidrug-resistant clinical isolates from a Moroccan tertiary-care hospital.

Methodology

A prospective laboratory-based study was conducted from March 1 to April 10, 2026. A total of 33 nonduplicate isolates were tested: *Klebsiella pneumoniae* (n = 15), *Escherichia coli* (n = 7), *Pseudomonas aeruginosa* (n = 5), *Acinetobacter baumannii* (n = 3), and methicillin-resistant *Staphylococcus aureus* (n = 3). Sodium hypochlorite 0.5%, hydrogen peroxide 3%, and ethanol 70% working solutions were serially diluted twofold with sterile water before inoculation. Subsequently, 50 µL of each prepared dilution was mixed 1:1 with 50 µL of bacterial suspension. Consequently, the highest final in-well biocide concentrations were 0.25% sodium hypochlorite, 1.5% hydrogen peroxide, and 35% ethanol. The estimated final bacterial inoculum was 5 × 10^5^ CFU/mL. After continuous broth exposure at 37°C for 18-24 hours, growth was assessed visually by turbidity and/or a bacterial pellet.

Results

A total of 30 isolates (90.9%) were recovered from hospitalized patients. No visible growth was observed for any isolate at the highest test condition (prepared working solutions of 0.5% sodium hypochlorite, 3% hydrogen peroxide, and 70% ethanol; final in-well concentrations 0.25%, 1.5%, and 35%, respectively). With sodium hypochlorite, growth occurred in 10/33 (30.3%) isolates at the 1/2 pre-inoculation dilution (0.125% final in-well), and all isolates grew at 1/8 (0.03125% final). Hydrogen peroxide suppressed visible growth through 1/16 (0.09375% final); growth occurred in 10/33 isolates at 1/32 (0.046875% final), and all isolates grew at 1/128. Ethanol suppressed visible growth through 1/8 (4.375% final); 27/33 isolates (81.8%) grew at 1/16 (2.1875% final), and all isolates grew at 1/32.

Conclusions

Under this prolonged broth model, the three biocides inhibited visible growth at the highest test conditions, while serial dilution produced a product-specific loss of inhibitory activity. Because equal volumes of biocide solution and inoculum were mixed, final in-well concentrations were half the prepared product concentrations. These findings support strict adherence to validated preparation and use procedures but do not define operational disinfectant concentrations, bactericidal surface efficacy, or clinical biocide resistance.

## Introduction

Antimicrobial resistance is a major threat to modern healthcare. The 2024 World Health Organization (WHO) bacterial priority pathogens list places carbapenem-resistant *Acinetobacter baumannii* and carbapenem-resistant Enterobacterales in the critical-priority group and carbapenem-resistant *Pseudomonas aeruginosa* in the high-priority group [1]. Healthcare-associated infections and antimicrobial resistance cause substantial preventable harm to patients and healthcare workers, and the WHO 2024 global report emphasizes that effective infection prevention and control programs, supported by basic water, sanitation, and hygiene services, can prevent a large proportion of these events [2].

Multidrug-resistant organisms may persist in the hospital environment and contaminate high-touch surfaces, shared equipment, wet reservoirs, and healthcare workers’ hands. Environmental persistence is particularly relevant for carbapenem-resistant Enterobacterales, *Acinetobacter baumannii*, and *Pseudomonas aeruginosa*, for which contaminated rooms and equipment have been linked to transmission and outbreaks [3]. Environmental cleaning and disinfection are therefore essential components of multimodal infection prevention, complementing hand hygiene, isolation precautions, surveillance, and antimicrobial stewardship.

Biocides used in healthcare include antiseptics applied to living tissue and disinfectants applied to inanimate surfaces. Sodium hypochlorite acts mainly through oxidative damage and chlorination of cellular components, hydrogen peroxide generates reactive oxygen species, and ethanol disrupts membranes and denatures proteins [4,5]. Although these agents have broad antimicrobial activity, their effectiveness depends on the applied concentration, contact time, organic load, temperature, pH, surface characteristics, product formulation, and correct preparation of working solutions [4-7]. Biofilm growth can further reduce susceptibility by limiting penetration and creating physiologically heterogeneous bacterial populations; standardized carrier studies have shown that activity varies according to the active ingredient, organism, and test conditions [6].

Reduced susceptibility to biocides has been described in several bacterial species and may involve intrinsic barriers, biofilm formation, reduced permeability, enzymatic degradation, stress responses, and efflux pumps [7-11]. Repeated exposure to subinhibitory concentrations may permit survival and adaptation, but interpretation remains difficult because standardized clinical breakpoints are unavailable and in-use disinfectant concentrations are generally much higher than laboratory inhibitory values [8,9]. Accordingly, this study uses the terms “visible growth at diluted concentrations” and “loss of inhibitory activity after dilution” rather than claiming clinical resistance to correctly used disinfectants.

Published Moroccan data evaluating hospital biocides against contemporary multidrug-resistant clinical isolates remain limited. A previous study from the same institution assessed antiseptics and disinfectants against 81 clinical and environmental *Acinetobacter baumannii* isolates and found that products were effective at their recommended or undiluted concentrations, whereas dilution reduced activity [12]. However, evidence covering multiple multidrug-resistant species and the three active agents evaluated together remains scarce. Accordingly, this exploratory pilot study aimed to characterize visible growth inhibition produced by hospital working solutions of sodium hypochlorite 0.5%, hydrogen peroxide 3%, and ethanol 70% across twofold serial dilutions against selected multidrug-resistant clinical isolates recovered at Mohammed V Military Teaching Hospital in Morocco. The study was specifically designed as a broth growth inhibition experiment; it was not intended to determine bactericidal activity, surface disinfection efficacy, clinical biocide resistance, or the operational disinfectant concentrations required to control antimicrobial resistance in hospital practice.

## Materials and methods

Study design and setting

This prospective, laboratory-based exploratory study was conducted from March 1 to April 10, 2026, in the Department of Clinical Bacteriology at Mohammed V Military Teaching Hospital, Rabat, Morocco. It evaluated the in vitro inhibitory activity of three biocides routinely used in hospital practice against selected clinical bacterial isolates recovered during routine diagnostic activity.

The study was designed as a pilot assessment using a purposive panel of clinically relevant isolates with multidrug-resistant phenotypes. No formal sample size calculation was performed because the aim was exploratory rather than prevalence estimation. The objective was to generate preliminary local data and describe whether progressive twofold dilution of the tested hospital working solutions permitted visible bacterial growth under a prolonged broth exposure model. The primary descriptive endpoint was the presence or absence of visible growth at each tested dilution.

Bacterial isolates and characterization

A total of 33 nonduplicate isolates were included: *Klebsiella pneumoniae* (n = 15; 45.5%), *Escherichia coli* (n = 7; 21.2%), *Pseudomonas aeruginosa* (n = 5; 15.2%), *Acinetobacter baumannii* (n = 3; 9.1%), and methicillin-resistant *Staphylococcus aureus* (MRSA; n = 3; 9.1%). Isolates were recovered from urine, respiratory samples, superficial pus, and blood cultures. Wild-type susceptible isolates and organisms considered nonpathogenic contaminants were excluded.

Species identification was performed by matrix-assisted laser desorption/ionization-time of flight mass spectrometry using the VITEK MS Prime system (bioMérieux, Marcy-l’Étoile, France). Antimicrobial susceptibility testing was conducted by the disk diffusion method on Mueller-Hinton agar, and results were interpreted according to the European Committee on Antimicrobial Susceptibility Testing 2025 guidelines. Based on their phenotypic resistance profiles, isolates were classified as carbapenemase producers, extended-spectrum beta-lactamase producers, high-level cephalosporinase producers, or MRSA. Molecular confirmation of the underlying resistance mechanisms was not routinely performed.

Biocide products and dilution series

The products tested were sodium hypochlorite 0.5%, hydrogen peroxide 3%, and ethanol 70%, corresponding to nominal hospital working solution concentrations used in healthcare practice [4]. The sodium hypochlorite product was labeled “Eau de Javel” (SCE Chemicals, Morocco; lot number and expiry date not stated on the available product label). The hydrogen peroxide 3% product was labeled “Eau oxygénée” (Nouvelles Pharma, Morocco; lot 12 302; expiry December 2029), and the ethanol 70% product was labeled “Alcool” (PRODISPHAR, Morocco; lot 451234; expiry December 2027). Additional formulation or additive details beyond the labeled active ingredient concentrations were not available from the product labels.

All dilution steps and plate inoculations were performed aseptically using sterile materials in sterile U-bottom 96-well microplates. Sterile water was used as the diluent for all three biocides. Twofold serial dilutions were prepared from each nominal working solution: working concentration (WC), 1/2, 1/4, 1/8, 1/16, 1/32, 1/64, 1/128, 1/256, and 1/512. Dilution labels used throughout the manuscript refer to the prepared product dilution before inoculation. In each test well, 50 µL of the prepared biocide dilution was mixed with 50 µL of bacterial suspension. Because the product and inoculum were mixed in equal volumes, the final in-well concentration of each biocide was one-half of its prepared pre-inoculation concentration. Thus, the WC wells contained final concentrations of 0.25% sodium hypochlorite, 1.5% hydrogen peroxide, and 35% ethanol.

Broth microdilution inhibitory assay

Isolates were subcultured on cystine lactose electrolyte-deficient agar and incubated at 37°C for 18-24 hours. Fresh colonies were suspended and adjusted by visual comparison to a 0.5 McFarland turbidity standard, corresponding approximately to 1 × 10^8^ CFU/mL. No automated turbidimeter or viable colony count was used to verify the starting suspension. The suspension was then diluted 1:100 in sterile Mueller-Hinton broth to obtain an estimated concentration of approximately 1 × 10^6^ CFU/mL.

In sterile U-bottom 96-well microplates, 50 µL of each prepared biocide dilution was mixed with 50 µL of bacterial suspension, yielding an estimated final bacterial inoculum of 5 × 10^5^ CFU/mL and halving the prepared biocide concentration in the test well. Positive-growth controls contained bacterial inoculum without biocide, and sterility controls contained broth and biocide without bacterial inoculum. The experimental design did not include a standardized organic-load challenge. The pH of the test mixtures was neither measured nor adjusted. Temperature was fixed at 37°C and was not evaluated as an independent experimental variable. Because no neutralization step was used, biocide exposure was continuous throughout the 18-24-hour aerobic incubation; no alternative short contact times were tested.

After incubation, each well was examined visually against a light background. Visible growth was defined as any detectable turbidity and/or a distinct bacterial pellet at the bottom of the U-shaped well relative to the sterility control; absence of visible growth required a clear well without a visible pellet, comparable with the sterility control. Positive-growth and sterility controls served as visual references. Any ambiguous well was re-examined side-by-side with both controls, and no unresolved discordant visual interpretations were recorded. For descriptive purposes, the operational inhibitory endpoint was the lowest tested dilution showing complete absence of visible growth. No spectrophotometric optical density threshold or automated turbidimetric reading was used.

No neutralization step, subculture from clear wells, minimum bactericidal concentration determination, or quantitative log-reduction assay was performed. The experiment therefore assessed inhibition of visible growth in broth and was not a standardized quantitative suspension, carrier, or dry-surface disinfectant efficacy test. The 18-24-hour interval represents prolonged continuous broth exposure rather than a practical disinfectant contact time. The assay did not assess bacterial killing, eradication, or real-world surface disinfection performance.

Data analysis

Data were entered and analyzed using Microsoft Excel (Microsoft Corp., Redmond, WA, USA). Results are reported as counts, percentages, and descriptive summaries. Because of the exploratory design and the small number of isolates within each species and resistance phenotype group, no inferential statistical comparisons were performed.

Ethical considerations

The study used bacterial isolates obtained during routine diagnostic procedures and minimal anonymized demographic data. No additional patient sampling, intervention, or follow-up was performed.

## Results

Characteristics of the isolates

The mean patient age was 65.3 years (range = 1 month to 92 years). The sex distribution was nearly balanced, with a slight male predominance (sex ratio = 1.06). Thirty (90.9%) isolates were recovered from hospitalized patients, and three (9.1%) were classified as community-origin isolates in the study records. Medical intensive care and emergency units contributed the largest proportions, accounting for 30.3% and 18.2% of isolates, respectively.

**Figure 1:**
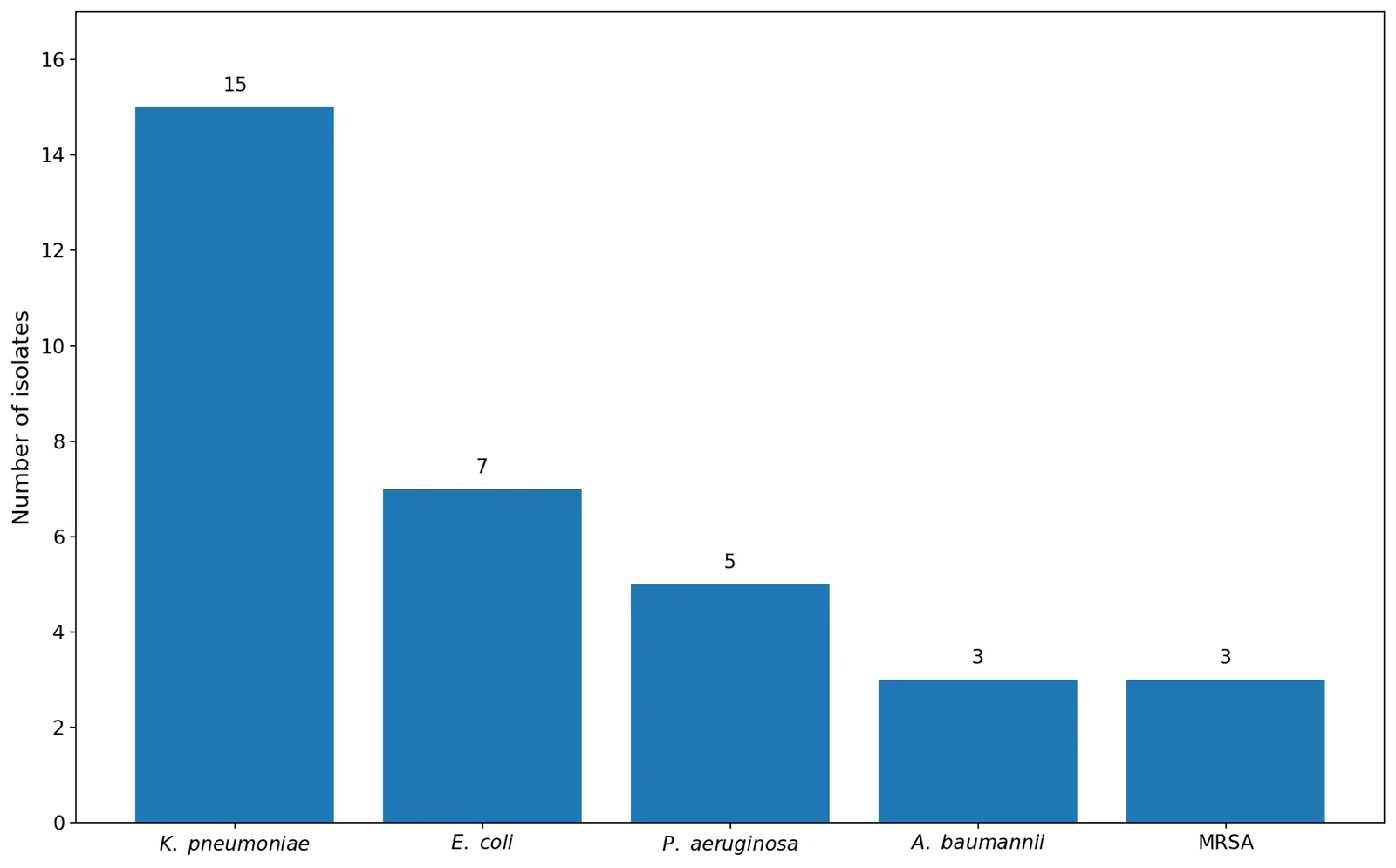
Distribution of MDR clinical isolates according to bacterial species. MDR = multidrug-resistant; MRSA = methicillin-resistant *Staphylococcus aureus*

Urine was the main specimen source (18/33; 54.5%), followed by respiratory samples (9/33; 27.3%), superficial pus (5/33; 15.2%), and blood culture (1/33; 3.0%). *Klebsiella pneumoniae* was the most frequent species and was recovered mainly from urine and respiratory samples. The isolate characteristics and species distribution are summarized in Table 1 and Figure 1.

**Table 1:** Main characteristics of the 33 MDR bacterial isolates. MDR = multidrug-resistant; ESBL = extended-spectrum beta-lactamase; MRSA = methicillin-resistant *Staphylococcus aureus*

Variable	Category	n (%)
Origin	Hospital-acquired isolates	30 (90.9)
	Community isolates	3 (9.1)
Species	Klebsiella pneumoniae	15 (45.5)
	Escherichia coli	7 (21.2)
	Pseudomonas aeruginosa	5 (15.2)
	Acinetobacter baumannii	3 (9.1)
	Methicillin-resistant *Staphylococcus aureus*	3 (9.1)
Specimen	Urine	18 (54.5)
	Respiratory samples	9 (27.3)
	Superficial pus	5 (15.2)
	Blood culture	1 (3.0)
Resistance phenotype	Carbapenemase-producing isolates	19 (57.6)
	ESBL producers	9 (27.3)
	High-level cephalosporinase producers	2 (6.1)
	MRSA	3 (9.1)

Sodium hypochlorite

**Figure 2:**
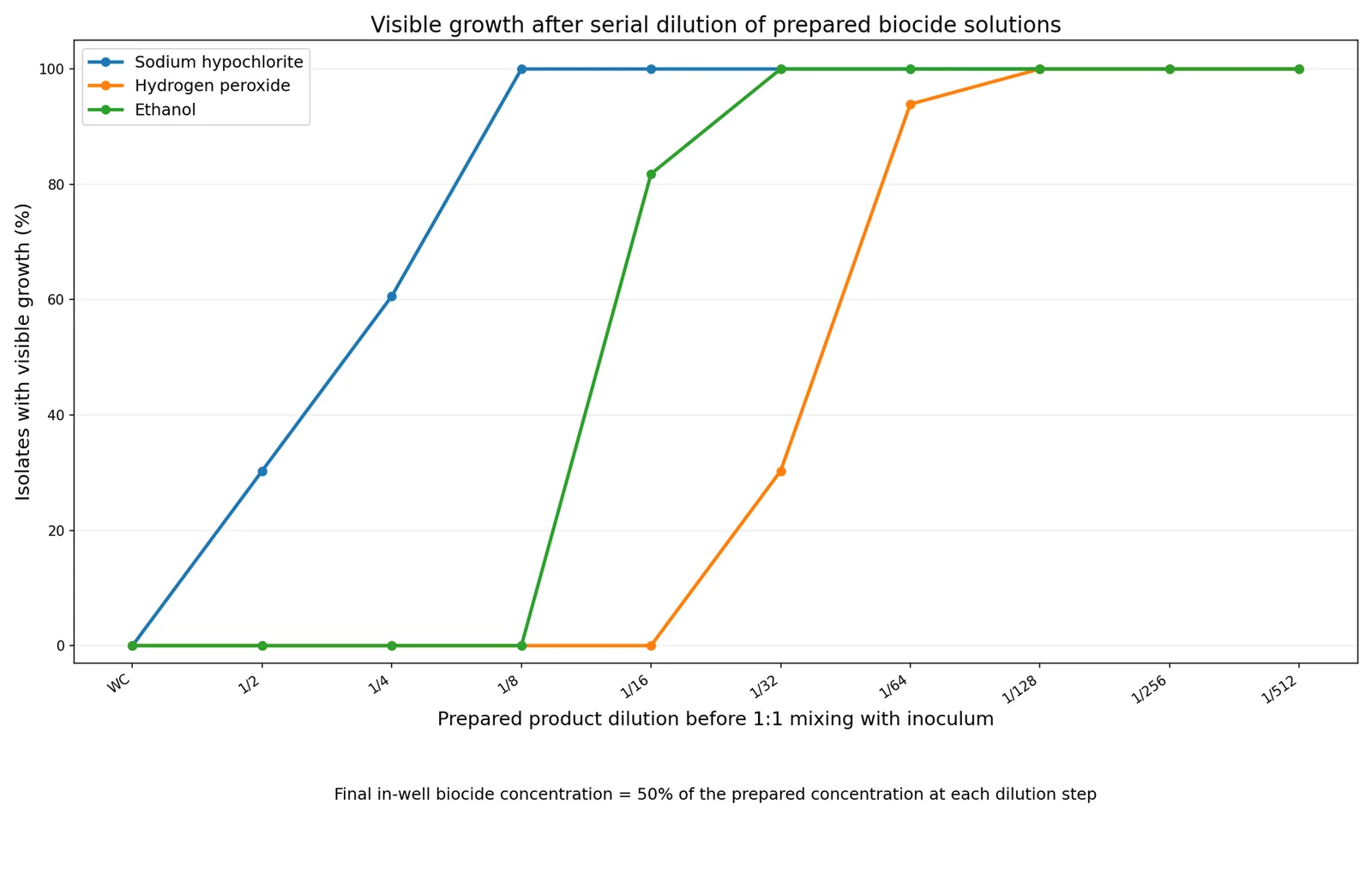
Percentage of MDR isolates showing visible growth after exposure to serial two-fold dilutions of the tested biocides. Dilution labels refer to prepared pre-inoculation product dilutions. Because each test well contained 50 µL of product dilution mixed with 50 µL of bacterial suspension, the final in-well biocide concentration was half of the prepared concentration at each step. MDR = multidrug-resistant

No visible growth was observed in wells receiving the highest prepared sodium hypochlorite solution (0.5% before inoculation; 0.25% final in-well). At the 1/2 pre-inoculation dilution (0.25% prepared; 0.125% final in-well), growth was observed in 10 (30.3%) isolates, including two of three MRSA isolates, two of five *Pseudomonas aeruginosa* isolates, and one of three *Acinetobacter baumannii* isolates. At 1/4 (0.0625% final in-well), 20 (60.6%) isolates showed visible growth. All isolates grew at 1/8 (0.03125% final in-well) and at higher dilutions (Table 2, Figure 2).

**Table 2:** Summary of visible growth after exposure to serial biocide dilutions. WC = working concentration

Biocide	Prepared working solution (pre-inoculation)	All isolates inhibited through	First dilution with visible growth	Dilution from which all isolates grew
Sodium hypochlorite	0.5% (0.25% final in-well)	WC (0.25% final)	1/2 (0.125% final; 10/33; 30.3%)	1/8 (0.03125% final)
Hydrogen peroxide	3% (1.5% final in-well)	1/16 (0.09375% final)	1/32 (0.046875% final; 10/33; 30.3%)	1/128 (0.01172% final)
Ethanol	70% (35% final in-well)	1/8 (4.375% final)	1/16 (2.1875% final; 27/33; 81.8%)	1/32 (1.09375% final)

Hydrogen peroxide

No visible growth was observed in wells receiving the highest prepared hydrogen peroxide solution (3% before inoculation; 1.5% final in-well) through the 1/16 pre-inoculation dilution (0.09375% final in-well). At 1/32 (0.046875% final in-well), growth occurred in 10 (30.3%) isolates, including three of five *Pseudomonas aeruginosa* isolates, five of 15 *Klebsiella pneumoniae* isolates, and one of three *Acinetobacter baumannii* isolates. At 1/64 (0.0234375% final in-well), 31 (93.9%) isolates showed growth, and all isolates grew from 1/128 onward (Table 2, Figure 2).

Ethanol

No visible growth was observed in wells receiving the highest prepared ethanol solution (70% before inoculation; 35% final in-well) through the 1/8 pre-inoculation dilution (4.375% final in-well). At 1/16 (2.1875% final in-well), 27 (81.8%) isolates showed growth. All isolates grew at 1/32 (1.09375% final in-well) and at higher dilutions (Table 2, Figure 2).

## Discussion

This pilot study provides local in vitro data on three commonly used hospital biocide working solutions tested against selected multidrug-resistant clinical isolates from a Moroccan tertiary-care hospital. Because 50 µL of each prepared product dilution was mixed with 50 µL of inoculum, the highest actual in-well concentrations were 0.25% sodium hypochlorite, 1.5% hydrogen peroxide, and 35% ethanol. Under the prolonged 18-24-hour broth exposure model, no visible growth occurred at these highest test conditions. Serial dilution was followed by a progressive, product-specific loss of inhibitory activity, with hydrogen peroxide maintaining inhibition across more dilution steps than the other products.

The practical relevance of these findings concerns infection prevention and control quality assurance rather than the definition of an effective operational concentration. In routine practice, disinfectants may be prepared incorrectly, inadvertently diluted, stored beyond validated periods, or applied for an insufficient contact time. The concentration-dependent loss of visible growth inhibition observed after serial dilution in this broth model reinforces the need for controlled preparation, storage, and staff training. However, these data cannot be used to derive surface-disinfection concentration thresholds because the assay used continuous broth exposure for 18-24 hours rather than a validated short contact time on a carrier or surface.

For sodium hypochlorite, no visible growth occurred at the highest test condition (0.5% prepared; 0.25% final in-well), whereas growth appeared after further dilution and was universal at the 1/8 pre-inoculation dilution (0.03125% final in-well). Chlorine-based products are broad-spectrum oxidizing agents, but their performance can be reduced by organic material and biofilm structures [4,13]. This is relevant to healthcare environments, where cleaning to remove organic soil should precede disinfection. Because organic load and short contact times were not evaluated in the present assay, these broth findings cannot be used to define an operational hypochlorite concentration.

Hydrogen peroxide produced the most sustained inhibition across the serial dilution range: visible growth first appeared at the 1/32 pre-inoculation dilution (0.046875% final in-well) and became nearly universal at 1/64. Oxidative stress defenses, including catalase and peroxidase activity, and biofilm-associated protection may influence bacterial responses to hydrogen peroxide [14]. The apparent pattern observed for *Pseudomonas aeruginosa* should be interpreted cautiously because only five isolates of this species were included and no standardized short contact time or organic load conditions were tested.

Ethanol showed no visible growth through the 1/8 pre-inoculation dilution, corresponding to 4.375% ethanol in the well after 1:1 mixing, followed by a sharp increase in growth at 1/16. This finding reflects prolonged broth exposure and must not be interpreted as evidence that such low ethanol concentrations are effective for routine disinfection. Alcohol-based products are generally most effective within an appropriate concentration range of approximately 60%-80%, because water contributes to protein denaturation [15]. Operational use should therefore follow the validated product concentration, volume, and contact time rather than the inhibitory endpoints observed in this experimental model.

Terminology is important in biocide studies. Unlike antimicrobial susceptibility testing for therapeutic antibiotics, universally accepted clinical breakpoints for disinfectant susceptibility are unavailable, and in-use concentrations are typically far above laboratory inhibitory endpoints [8,9]. The present findings therefore do not demonstrate clinical resistance to sodium hypochlorite, hydrogen peroxide, or ethanol. They show visible growth at selected serial dilutions under the specific conditions of a nonstandardized broth assay.

This study has several limitations. The sample was small, purposively selected, and enriched for multidrug-resistant phenotypes; it did not include a susceptible comparator group and cannot estimate the prevalence of altered biocide susceptibility. The assay measured visible growth inhibition after continuous 18-24-hour broth exposure at 37°C and did not reproduce standardized short contact times, dry or semi-dry surfaces, clean versus dirty conditions, standardized organic soil, blood, secretions, biofilm, or different surface materials. The pH was neither measured nor adjusted, and temperature was fixed rather than evaluated as an experimental factor. The starting inoculum was adjusted by visual comparison with a 0.5 McFarland standard and estimated from the subsequent dilution factor; no automated turbidity measurement or viable count confirmation was performed. Endpoints were also read visually from turbidity and/or pellet formation without spectrophotometric optical density measurements. Although ambiguous wells were rechecked against positive-growth and sterility controls and no unresolved discordant visual interpretations were recorded, visual reading remains less objective than instrumental measurement. No neutralization, minimum bactericidal concentration, quantitative log-reduction, or postexposure viability assessment was performed. Consequently, bacterial killing and real-world disinfectant efficacy were not established. Molecular determinants of antimicrobial resistance and biocide tolerance were not evaluated, and the small species-specific groups precluded inferential comparisons.

Future studies should use larger and more diverse clinical and environmental isolate collections, include susceptible comparator organisms, and apply standardized quantitative suspension and carrier/surface methods under clean and dirty conditions. Defined short contact times, validated neutralization, quantitative log-reduction endpoints, replicate testing, viable count confirmation of inoculum density, spectrophotometric or automated growth measurement, controlled pH conditions, temperature documentation, and molecular screening for tolerance-associated determinants would provide more objective, reproducible, and clinically interpretable evidence.

## Conclusions

In this exploratory broth assay, sodium hypochlorite, hydrogen peroxide, and ethanol inhibited visible growth of all tested multidrug-resistant clinical isolates at the highest test conditions. Equal-volume mixing with the bacterial inoculum reduced the final in-well biocide concentrations to one-half of the prepared concentrations (0.25%, 1.5%, and 35%, respectively). Serial dilution produced a progressive and product-specific loss of inhibitory activity. The results support strict adherence to validated product concentrations, preparation procedures, storage conditions, and contact times. Because the study used prolonged continuous broth exposure, visual growth endpoints, and no neutralization or standardized surface-based method, the findings do not define the concentrations required for hospital control of antimicrobial resistance and should not be interpreted as proof of real-world disinfection performance or clinical biocide resistance.
